# Design of Selective Detector for Distributed Targets Through Stochastic Characteristic of the Fictitious Signal

**DOI:** 10.3390/s25030736

**Published:** 2025-01-25

**Authors:** Gaoqing Xiong, Hui Cao, Weijian Liu, Jialiang Zhang, Kehao Wang, Kai Yan

**Affiliations:** 1 School of Information Engineering, Wuhan University of Technology, Wuhan 430070, China; 288520@whut.edu.cn (G.X.); kehao.wang@whut.edu.cn (K.W.); 2 Hubei Key Laboratory of Broadband Wireless Communication and Sensor Networks, Wuhan University of Technology, Wuhan 430070, China; 3 Wuhan Electronic Information Institute, Wuhan 430019, China; liuvjian@163.com (W.L.); yankai_kevin@163.com (K.Y.); 4 Electrical and Electronics Dream Factory, Wuhan 430070, China

**Keywords:** distributed targets, signal mismatch, fictitious signal, constant false alarm rate

## Abstract

We investigate the problem of detecting the distributed targets buried in the Gaussian noise whose covariance matrix is unknown when signal mismatch occurs. The idea is to add a fictitious signal under the null hypothesis of the origin detection problem so that when signal mismatch occurs, the fictitious signal captures the mismatched signals, thus making the null hypothesis more plausible. More precisely, the fictitious signal is modeled as a Gaussian component with a covariance matrix of a stochastic factor multiplied by a rank-one matrix. The generalized likelihood ratio test (GLRT) is employed to address the modification detection problem. We present an exhaustive derivation of the detector and prove that it possesses the constant false alarm rate (CFAR) property. The performance analysis demonstrates the effectiveness of the proposed detector. When the SNR is 23 dB, as generalized cosine squared decreases from 1 to 0.83, the detection probability of the proposed GLRT-SL drops to 0.65, exhibiting the fastest decline compared to the G-ABORT-HE, which falls to 0.98, and the GW-ABORT-HE, which decreases to 0.85.

## 1. Introduction

The primary mission of a radar system is to detect and track targets of interest within an intricate environment rife with noise, clutter, and potential interference [1,2,3,4]. In essence, radar target detection is based on a detector. One would like a detector to deliver as high a probability of detection as possible under matched conditions, i.e., when the actual steering vector coincides with the nominal one, while minimizing errors. Also, it is usually desirable to have a detector with the constant false alarm (CFAR) property that allows the desired threshold to be set without considering the statistics of the noise. Naturally, a criterion called the generalized likelihood ratio test (GLRT) that meets the above two objectives was proposed for solving the problem of point-like target detection in Gaussian noise [5]. Kelly also derived the statistical distribution of the GLRT statistic under the signal-present hypothesis and the null hypothesis and conducted a performance analysis. It is worth nothing that any detector based on Kelly’s GLRT statistic naturally inherits the CFAR property and is more convenient for performance evaluation [6]. In addition, there are some other well-known detectors for the classical detection problem, such as adaptive match filter (AMF) [7] and De Maio’s Rao (DMRao) test [8]. The Wald test proposed in [9] has identical detection statistics to the AMF.

In practice, when the target is large or the radar resolution is high, the echoes reflected from the target could occupy multiple range cells, in which case the point-like target model becomes insufficient [10]. The target of this nature is commonly referred to as a distributed target. The problem of distributed target detection has been extensively studied. In [11], the GLRT and two-step GLRT for distributed targets embedded in Gaussian noise with an unknown covariance matrix were proposed. In [12], two-step variants of GLRT, Rao, and Wald criteria were employed to address the distributed target detection in compound-Gaussian sea clutter. In this work, for the purpose of reducing the amount of training data, the covariance matrix is assumed to possess a persymmetric structure, which is estimated utilizing the fixed-point estimator. The GLRT-based detectors were derived in [13] for distributed target detection in the presence of partially homogeneous noise plus subspace interference. Related studies on distributed MIMO radar can be found in [14,15,16]. It was mentioned in [17] that as radar resolution increases, distributed targets exhibit weaker perturbations compared to point-like targets, which is one of the factors that brings about performance improvement. It naturally follows that the number of unknowns in the classical problem model grows with the range cells. In contrast to the usual assumption of deterministic target amplitude in the classical problem, a target model with a constant number of unknowns is proposed in [18], which treats the target amplitude as a stochastic variable that follows a zero-mean Gaussian distribution with unknown variance. Specifically, the variance of the observation matrix under signal-present hypothesis is given by $\Sigma +Pvv^{H}$, where v is the target steering vector, Σ represents the noise covariance matrix and P is a positive stochastic variable. The stochastic variable P eliminates the drawback of having a large number of unknown amplitudes in classical distributed target models. The statistical distribution form of binary hypothesis test can be expressed as

(1) $$\left\{\begin{array}{l} H_{0}:X\sim CN_{N,K}\left(0,\Sigma ,I_{K}\right)\\ H_{1}:X\sim CN_{N,K}\left(0,\Sigma +Pvv^{H},I_{K}\right) \end{array}\right.$$

This newly derived GLRT performs better than the natural competitors under the usual target model for a small number of training data. Further research on this stochastic rank-one model can be found in [19,20,21,22].

The majority of the aforementioned research has been carried out with the underlying assumption of the absence of signal mismatch. However, signal mismatch inevitably occurs due to uncalibrated arrays, uncertainties about the direction of arrival of the target and the possible sidelobe interference, etc., [23,24,25]. Detectors can typically be categorized into two types based on their sensitivity against mismatched signals: selective detectors and robust detectors. The characteristic of a selective detector is that the detection probability decreases rapidly with the increase in mismatch. In contrast, a robust detector maintains a high probability of detection. In some practical scenarios, such as target orientation, it is more appropriate for the detector to possess some selectivity, whereas when the radar is operating in search mode, a robust detector is more desirable. Hence, many studies have investigated how to improve the selectivity or robustness of detectors against signal mismatch.

A common and dominant selective detector design technique is to add a fictitious signal under the null hypothesis and assume that this fictitious signal is orthogonal to the nominal steering vector in truly whitened space or quasi-whitened space [26,27]. When signal mismatch occurs, the whitened or quasi-whitened component of the mismatched signals in the direction of the fictitious signal causes the detector to prefer the null hypothesis and is less likely to declare a detection. Many selective detectors have been designed based on these ABORT-like idea [28,29,30,31]. In [28], the idea of W-ABORT was relaxed by introducing a Bernoulli random variable multiplied by a fictitious signal; thereby, an adjustable detector with an improved detection vs. rejection tradeoff was proposed. The promotion form of W-ABORT in a partially homogeneous environment was reported in [29]. In [30], the generalized ABORT was derived for the distributed targets, but its selectivity is not strong enough. To address the shortage of training samples, probabilistic modeling was adopted for fictitious signals in [31], and a Bayesian framework was introduced in the design phase. In [32], a selective detector called the double-normalized adaptive matched filter (DN-AMF) was proposed with the idea of assuming that both the signal-present hypothesis and null hypothesis contain noise-like interference and a suitable design criterion is used. It should be noted that this approach seems to lack a reasonable explanation and largely depends on a specific design criterion. For approaches to enhance robustness, see [33,34,35,36].

Notice that the ABORT-like idea is to model the fictitious signal injected under the null hypothesis as a deterministic unknown quantity. To the best of our knowledge, the approach to treating it as a stochastic quantity has not been considered. Inspired by the rank-one stochastic signal model in [18] and the ABORT-like idea, we investigate a possible approach to selective detector design for distributed targets. The proposed idea is to introduce a fictitious signal under the null hypothesis that obeys a complex Gaussian distribution with zero mean and variance of an unknown stochastic variable multiplied by a known rank-one matrix. The rank-one matrix is composed of the mismatched steering vector around the target steering vector. In fact, this model is a possible generalization of the fictitious signal introduced in [26,27] and the stochastic target model $Pvv^{H}$ in [18]. Furthermore, this fictitious signal does not exhibit any form of orthogonality with the target steering vector. When signal mismatch occurs, the leaked signal is precisely captured by the stochastic fictitious signal, which makes the null hypothesis more plausible. We derive the detector using the GLRT and show that it possesses the CFAR property. The proposed GLRT demonstrates the highest selectivity compared to its natural competitors when the fictitious steering vector is a slightly mismatched target steering vector.

## 2. Problem Formulation

Assume that the radar system receives N pulses in a pulse processing interval (CPI). The echoes reflected from the target occupy consecutive K range cells, which are usually referred to as the test data. The test data of the *k*th range cell are recorded as $x_{k}\in {\mathbb{C}}^{N\times 1}$, k=1,⋯,K. For a binary hypothesis test of an injected fictitious signal, under the null hypothesis, $x_{k}$ contains noise $n_{k}$ and the fictitious signal $\xi_{k}$. Conversely, under the hypothesis of the signal being present, $x_{k}$ is composed of the target signal and noise. Specifically, the structure of the target signal is expressed as $a_{k}^{*}h$, where $a_{k}^{*}$ stands for the unknown target amplitude and h denotes the known normalized target steering vector. Hence, the binary hypothesis test can be written as

(2) $$\left\{\begin{array}{l} H_{0}:x_{k}=\xi_{k}+n_{k};k=1,\ldots ,K\\ H_{1}:x_{k}=a_{k}^{*}h+n_{k};k=1,\ldots ,K \end{array}\right.$$

Further, we assume that the injected fictitious signal $\xi_{k}$, k=1,⋯,K is independently and identically distributed (IID) and distributed as $\xi_{k}\sim CN_{N}\left(0,Pdd^{H}\right)$, where P is an unknown positive factor, $d\in {\mathbb{C}}^{N\times 1}$ is a known column vector indicating the mismatched steering vector around the target steering vector h. The rank-one matrix $dd^{H}$ reflects our knowledge of the stochastic variations around h. When signal mismatch occurs, $dd^{H}$ collects some, if not the whole, of the leaked steering vector that is different from h, and the factor P captures the corresponding target amplitude. As a result, a detector is more inclined to decide the null hypothesis. In addition, the noise $n_{k}$ is usually assumed to have an IID complex Gaussian distribution with zero mean and variance of a positive definite matrix Σ, i.e., $n_{k}\sim CN_{N}\left(0,\Sigma \right)$. Here, we consider the case where Σ is unknown. To estimate the noise covariance matrix Σ, the training data collected in the vicinity of the test data is in used, which is usually assumed to contain only noise and share the statistical characteristics of the noise with the test data. Suppose there are L training data, which is denoted as $y_{e,l}$, l=1,⋯,L, then accordingly its distribution is written as $y_{e,l}\sim CN_{N}\left(0,\Sigma \right)$. Finally, assume that L>N. Based on the above assumptions, we summarize as

(3) $$\left\{\begin{array}{l} H_{0}:\left\{\begin{array}{l} X\sim CN_{N,K}\left(0,\Sigma +Pdd^{H},I_{K}\right)\\ Y\sim CN_{N,L}\left(0,\Sigma ,I_{L}\right) \end{array}\right.\\ H_{1}:\left\{\begin{array}{l} X\sim CN_{N,K}\left(ha^{H},\Sigma ,I_{K}\right)\\ Y\sim CN_{N,L}\left(0,\Sigma ,I_{L}\right) \end{array}\right. \end{array}\right.$$

where $X=\left[x_{1},\cdots ,x_{K}\right]\in {\mathbb{C}}^{N\times K}$, $Y=\left[y_{e,1},y_{e,2},\cdots ,y_{e,L}\right]\in {\mathbb{C}}^{N\times L}$, and $a={\left[a_{1},a_{2},\cdots ,a_{K}\right]}^{T}\in {\mathbb{C}}^{K\times 1}$.

*Notations*: Matrices are represented by bold uppercase letters, vectors by bold lowercase letters, and scalars by lowercase letters. ${\mathbb{C}}^{p\times q}$ stands for the complex matrix space with dimension p×q. For a matrix $\Sigma \in {\mathbb{C}}^{N\times N}$, $\mathrm{tr}\left\{\Sigma \right\}$, $\mathrm{etr}\left\{\Sigma \right\}$, and $\left|\Sigma \right|$ are the trace of Σ, the exponential of the trace of Σ, the determinant of Σ, respectively. ${\left(\cdot \right)}^{*}$, ${\left(\cdot \right)}^{T}$ and ${\left(\cdot \right)}^{H}$ represent conjugate, transpose and conjugate transpose, respectively. If Σ is a nonsingular matrix, $\Sigma^{-1}$ denotes its inverse. If Σ is a positive definite matrix, $\Sigma^{1/2}$ stands for its Hermitian square root. $I_{N}$ is an N×N identity matrix. $P_{h}=hh^{H}{\left(h^{H}h\right)}^{-1}$ is the orthogonal projection matrix onto the subspace spanned by N×1 column vector h, and $P_{h}^{⊥}=I_{N}-P_{h}$. $Z\sim CN_{N,K}\left(M,\Sigma_{A},\Sigma_{B}\right)$ denotes that the matrix follows the complex matrix variate Gaussian distribution with probability density function (PDF) $f\left(Z\right)=\pi^{-NK}{\left|\Sigma_{A}\right|}^{-K}{\left|\Sigma_{B}\right|}^{-N}\mathrm{etr}\left\{-{\left(Z-M\right)}^{H}\Sigma_{A}^{-1}\left(Z-M\right)\Sigma_{B}^{-1}\right\}$. As for the complex (vector) Gaussian distribution, we note as $z\sim CN_{N}\left(a,\Sigma_{a}\right)$, where z and a are the N×1 column vectors. $CW_{N}\left(K,\Sigma_{t}\right)$ stands for the N×N complex Wishart distribution with K degrees of freedom and scale matrix $\Sigma_{t}$.

## 3. Detector Design

The design criteria of GLRT for the detection problem in (2) can be expressed as

(4) $$t_{\mathrm{GLRT}}=\frac{\underset{\Sigma ,a}{\max}f_{1}\left(X,Y\right)}{\underset{\Sigma ,P}{\max}f_{0}\left(X,Y\right)}$$

where

(5) $$f_{1}\left(X,Y\right)=\pi^{-N\left(L+K\right)}{\left|\Sigma \right|}^{-\left(L+K\right)}\mathrm{etr}\left\{-\Sigma^{-1}\left[S+\left(X-ha^{H}\right){\left(X-ha^{H}\right)}^{H}\right]\right\}$$

and

(6) $$f_{0}\left(X,Y\right)=\pi^{-N\left(L+K\right)}{\left|\Sigma \right|}^{-L}{\left|\Sigma +Pdd^{H}\right|}^{-K}\mathrm{etr}\left\{-\Sigma^{-1}S-{\left(\Sigma +Pdd^{H}\right)}^{-1}XX^{H}\right\}$$

represent the joint PDF of X and Y under $H_{1}$ and $H_{0}$, respectively. S is defined as L times sample covariance matrix (SCM), i.e., $S=\sum_{l=1}^{L}y_{e,l}y_{e,l}^{H}=YY^{H}$.

The detailed derivation procedure can be found in Appendix A. Here, we directly present the final GLRT as

(7) $$t_{\mathrm{GLRT}}=t_{\mathrm{GLRT}\text{-}\mathrm{HE}}\times \underset{0<\mu <1}{\min}\mu^{-\frac{K}{L+K}}\left[\frac{d^{H}{\left(S+XX^{H}\right)}^{-1}d}{d^{H}{\left(S+\mu XX^{H}\right)}^{-1}d}\right]$$

where $t_{\mathrm{GLRT}\text{-}\mathrm{HE}}$ is the one-step GLRT for homogeneous environment, which is equivalent to Kelly’s GLRT when K=1. The statistic of the GLRT-HE is denoted as [11]

(8) $$t_{\mathrm{GLRT}\text{-}\mathrm{HE}}=\frac{\left|I_{K}+{\tilde{X}}^{H}\tilde{X}\right|}{\left|I_{K}+{\tilde{X}}^{H}P_{\tilde{h}}^{⊥}\tilde{X}\right|}$$

where $\tilde{X}=S^{-1/2}X$, $\tilde{h}=S^{-1/2}h$, $P_{\tilde{h}}=\tilde{h}{\tilde{h}}^{H}/\left({\tilde{h}}^{H}\tilde{h}\right)$, and $P_{\tilde{h}}^{⊥}=I_{N}-P_{\tilde{h}}$.

**Remark 1.**  *The GLRT statistic is constituted by the product of two terms. The first term is the GLRT-HE statistic, and the second term is a correction term arising from the stochastic nature of the injected fictitious signal. When the correction term approaches 1, it can be deduced that the estimate of* μ *is approximately equal to 1. In such a scenario,* d *exerts minimal influence on the GLRT, and the overall performance of the GLRT remains comparable to that of the GLRT-HE. In contrast, when* μ *tends to 0, under the assumption that* X *contains a strong target, the denominator of the correction term is more likely to eliminate it, and* d *will have a stronger effect on the selectivity of the GLRT. Note that* d≠h *and* d *are not related to* h *in any orthogonal form*.

**Remark 2.**  *The presence of the minimum optimization problem in the correction term introduces additional computational overhead for the GLRT compared to the GLRT-HE. However, this overhead is not considered to be significant. The correction term in (7) can be further expanded as* 

(9) $${\hat{\mu }}_{0}=\underset{0<\mu <1}{\min}\mu^{-\frac{K}{L+K}}\left[\frac{d^{H}S^{-1}d-d^{H}S^{-1}X{\left(I_{K}+X^{H}S^{-1}X\right)}^{-1}X^{H}S^{-1}d}{d^{H}S^{-1}d-\mu d^{H}S^{-1}X{\left(I_{K}+\mu X^{H}S^{-1}X\right)}^{-1}X^{H}S^{-1}d}\right]$$

*which can be efficiently computed by performing a grid search on* μ*. Another equivalent form of the GLRT is as follows* 

(10) $$t_{\mathrm{GLRT}}=\frac{\left|I_{K}+{\tilde{X}}^{H}P_{\tilde{d}}^{⊥}\tilde{X}\right|}{\left|I_{K}+{\tilde{X}}^{H}P_{\tilde{h}}^{⊥}\tilde{X}\right|}\times \underset{0<\mu <1}{\min}\mu^{-\frac{K}{L+K}}\frac{\left|I_{K}+\mu {\tilde{X}}^{H}\tilde{X}\right|}{\left|I_{K}+\mu {\tilde{X}}^{H}P_{\tilde{d}}^{⊥}\tilde{X}\right|}$$

*For the optimization problem in determinant-based GLRT, an efficient computation approach is to use the fact that* 

(11) $$\left|I_{K}+\mu M\right|=\prod_{j=1}^{K}\left[1+\mu \lambda_{j}\left(M\right)\right]$$

*where* $\lambda_{j}\left(M\right)$ *denotes the eigenvalues of* M.

**Remark 3.**  *The distribution of the GLRT statistic is independent of* Σ *under the null hypothesis, i.e., the GLRT is CFAR against* Σ. *For proof, see Appendix B*.

## 4. Performance Analysis

We employ Monte Carlo simulation to assess the behavior of the designed detector. The probability of false alarm (PFA) is fixed at $\mathrm{PFA}=10^{3}$. In order to determine the detection threshold and the probability of detection (PD), 100/PFA and $10^{4}$ independent trials are performed, respectively. For the covariance matrix, the standard exponentially correlated model is considered, where the $\left(i,j\right)$th element of Σ is denoted as $\varepsilon^{\left|i-j\right|}$, i,j=1,2,⋯,N, where ε is the one-lag correlation coefficient. For the training data Y, we construct it as $Y=\Sigma^{1/2}W_{L}$, where $W_{L}$ is a complex Gaussian white noise with N×L dimensions and variance of 1. Similarly, the noise term is generated by $\Sigma^{1/2}W_{K}$, where $W_{K}$ is a complex Gaussian white noise with N×L dimensions and variance of 1. The structure of the target steering vector is

(12) $$h=\frac{1}{\sqrt{N}}{\left[1,e^{-j2\pi \bar{f}},\cdots ,e^{-j2\pi \left(N-1\right)\bar{f}}\right]}^{T}$$

where j represents the imaginary unit and $\bar{f}$ is the normalized Doppler frequency. Under mismatched conditions, the normalized frequency of the actual steering vector $h_{0}$ is $\bar{f}+\delta /N$. To better quantify the degree of mismatch between h and $h_{0}$, the widely used generalized cosine squared (GCS) is introduced below with [26]

(13) $$\cos^{2}\theta =\frac{{\left|h_{0}^{H}\Sigma^{-1}h\right|}^{2}}{h_{0}^{H}\Sigma^{-1}h_{0}h^{H}\Sigma^{-1}h}$$

where θ represents the angle between the whitened versions of h and $h_{0}$. It is straightforward to conclude that δ=0 indicates no mismatch, corresponding to $\cos^{2}\theta =1$. Similarly, the mismatched steering vector d can be configured through $\bar{f}+\delta_{d}/N$, with its GCS defined as

(14) $$\cos^{2}\phi =\frac{{\left|d^{H}\Sigma^{-1}h\right|}^{2}}{d^{H}\Sigma^{-1}dh^{H}\Sigma^{-1}h}$$

where ϕ represents the angle between the whitened versions of h and d. The signal to noise ratio (SNR) is defined as

(15) $$\mathrm{SNR}=a^{H}ah_{0}^{H}\Sigma^{-1}h_{0}$$

For comparison, the generalized ABORT and its whitened version in homogeneous environment are considered due to their ability to reject mismatched signals. In the subsequent simulations, these two detectors are recorded as G-ABORT-HE [30] and GW-ABORT-HE [28], respectively. The detection statistics of these two selective detectors are given by

(16) $$t_{G\text{-}\mathrm{ABORT}\text{-}\mathrm{HE}}=\frac{\left|I_{K}+{\tilde{X}}^{H}P_{\tilde{h}}\tilde{X}\right|}{\left|I_{K}+{\tilde{X}}^{H}P_{\tilde{h}}^{⊥}\tilde{X}\right|}$$

and

(17) $$t_{\mathrm{GW}\text{-}\mathrm{ABORT}\text{-}\mathrm{HE}}=\frac{\left|I_{K}+{\tilde{X}}^{H}\tilde{X}\right|}{{\left|I_{K}+{\tilde{X}}^{H}P_{\tilde{h}}^{⊥}\tilde{X}\right|}^{2}}$$

respectively. Additionally, since the proposed GLRT includes the GLRT-HE statistic, the GLRT-HE is also introduced [11], primarily for comparison of matched detection performance. Unless otherwise specified, the settings N=16, $\bar{f}=0.08$, and ε=0.9 are consistently applied.

**Figure 1:**
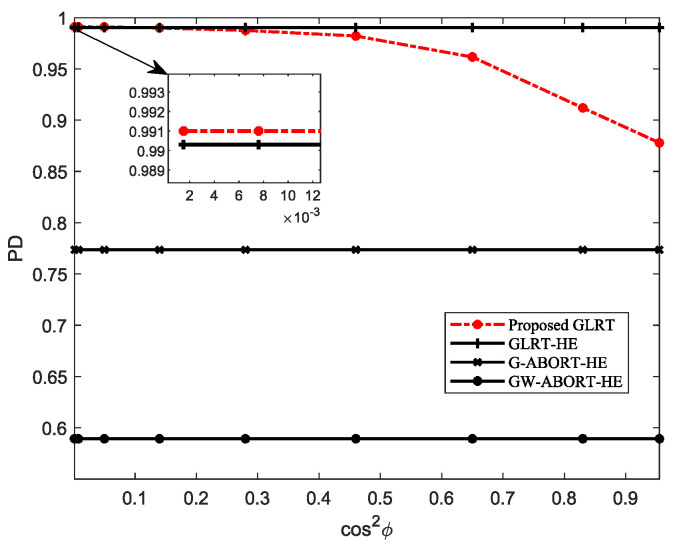
PD versus $\cos^{2}\phi$ for N=16, L=2N, K=4 and SNR=18 dB.

Figure 1 depicts the PD of the proposed GLRT under different d. The result indicates that as the degree of mismatch of d decreases (i.e., $\cos^{2}\phi$ increases), the PD of the proposed GLRT initially exhibits a gradual decline, followed by a rapid drop. Compared to the GLRT-HE, the proposed GLRT exhibits a certain performance loss when $\cos^{2}\phi$ is large, whereas it has a similar PD as the GLRT-HE or even a slight performance gain when $\cos^{2}\phi$ is small. Regardless of what degree d is the mismatched steering vector around h, the proposed GLRT consistently outperforms the G-ABORT-HE and GW-ABORT-HE. In view of the above results, in subsequent simulations, we then consider three scenarios for d: slight mismatch, moderate mismatch, and severe mismatch, labeled as GLRT-SL, GLRT-MO, and GLRT-SE, respectively. More specifically, we select $\delta_{d}=0.2$, 0.4 and 0.6, corresponding to $\cos^{2}\phi =0.83$, 0.46, and 0.14.

**Figure 2:**
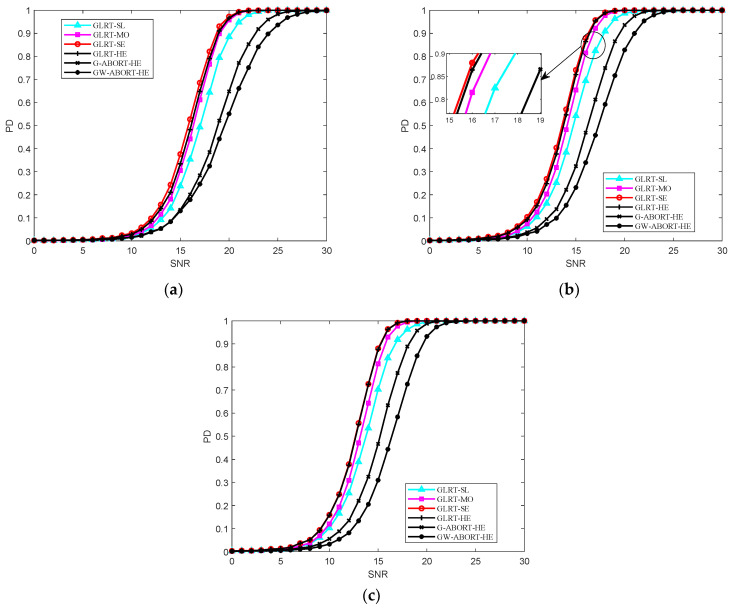
PD versus SNR for N=16, K=4 and $\cos^{2}\theta =1$: (**a**) L=1.5N; (**b**) L=2N; (**c**) L=2.5N.

Figure 2 reports the PD versus SNR under matched conditions. It is clear that the GLRT-SE has the best behavior at L=1.5N, with a slight performance overrun compared to the GLRT-HE. As L increases to 2.5N, the PD of the GLRT-SE and GLRT-HE becomes nearly identical. The PD of GLRT-MO is between that of the GLRT-SL and GLRT-SE, and the GLRT-SE is more powerful than the G-ABORT-HE and GW-ABORT-HE. The findings align with those depicted in Figure 1.

**Figure 3:**
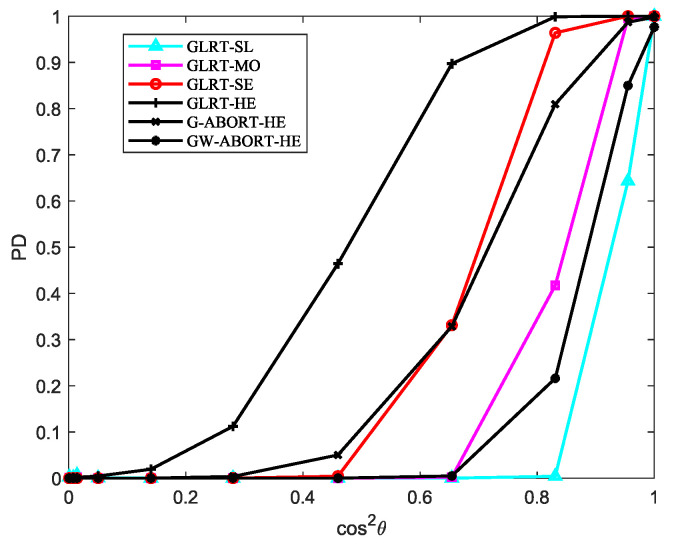
PD versus $\cos^{2}\theta$ for N=16, L=2N, K=4 and SNR=23 dB.

The PD versus $\cos^{2}\theta$ for SNR=23 dB is reported in Figure 3. As observed, as the degree of signal mismatch intensifies, the GLRT-SL exhibits the fastest decline, indicating that it has the strongest selectivity compared to its natural competitors. The GLRT-MO demonstrates greater selectivity than the G-ABORT-HE but is less selective than the GW-ABORT-HE. For the GLRT-SE, the decrease in PD is slower than that of the G-ABORT-HE when $\cos^{2}\theta >0.65$ (corresponding to δ=0.3), and the GLRT-SE no longer provides a PD above 0.5 when $\cos^{2}\theta \leq 0.65$. Therefore, the selectivity of GLRT-SE is inferior to that of the G-ABORT-HE. Notably, compared to the other two scenarios, considering d as a slightly mismatched steering vector around h enhances the ability to capture mismatched signals. As a result, when signal mismatch occurs, the detector is more likely to favor the null hypothesis, leading to the fastest rate of decline observed in the figure.

**Figure 4:**
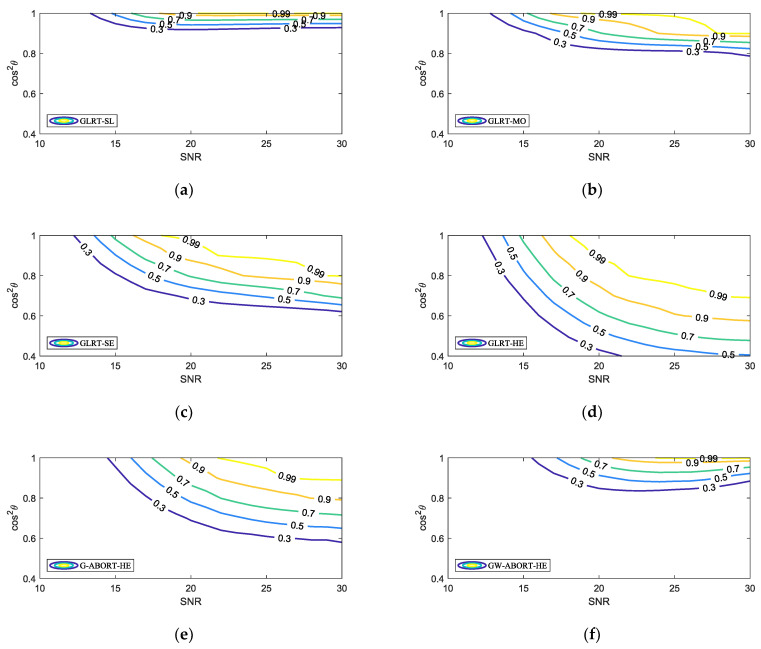
Contour of the PD versus SNR and $\cos^{2}\theta$ for N=16, L=2N and K=4: (**a**) GLRT-SE; (**b**) GLRT-MO; (**c**) GLRT-SE; (**d**) GLRT-HE; (**e**) G-ABORT-HE; (**f**) GW-ABORT-HE.

Figure 4 further presents the selectivity of the proposed GLRT against mismatched signals. The mesa plot was first depicted in [26] to describe the PD of a detector under different SNR and $\cos^{2}\theta$. Along a fixed SNR, the faster the PD decreases as $\cos^{2}\theta$ diminishes, the stronger the selectivity of a detector against mismatched signals. Overall, as the mismatch increases, the rate of PD decline, from fastest to slowest, follows the order of the GLRT-SL, GW-ABORT-HE, GLRT-MO, G-ABORT-HE, GLRT-SE, and GLRT-HE. This result is fully consistent with that observed in Figure 3. Note that among the detectors corresponding to the three cases of d, the GLRT-SL exhibits the strongest selectivity but has the lowest PD for matched targets, while the GLRT-SE is the most powerful but less selective compared to the other two. This indicates that setting d as a smaller mismatched steering vector around h enhances selectivity at the cost of sacrificing matched detection performance. Moreover, the GLRT-SL outperforms the GW-ABORT-HE in both selectivity and matched detection performance, and the GLRT-MO similarly outperforms the G-ABORT-HE. These results highlight the advantage of the approach outlined in model (2) over the ABORT-like idea in designing selective detectors for distributed targets.

**Figure 5:**
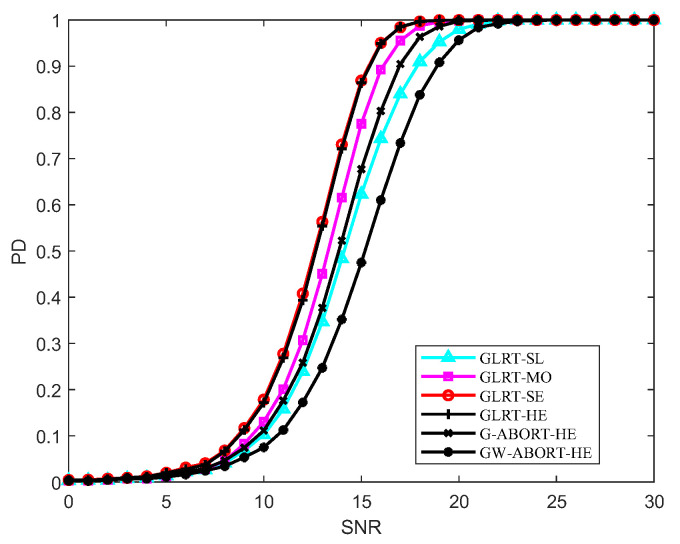
PD versus SNR for N=16, L=2N, and K=2.

**Figure 6:**
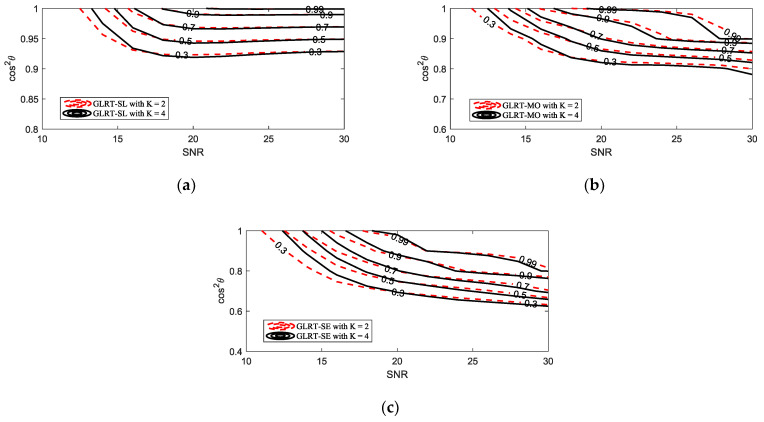
Comparison contours of the PD versus SNR and $\cos^{2}\theta$ for N=16 and L=2N: (**a**) GLRT-SL; (**b**) GLRT-MO; (**c**) GLRT-SE.

In order to gain further insight into the behavior of the proposed GLRT, we consider a lower value of K. The PD versus SNR for K=2 under matched conditions is reported in Figure 5, and the comparison mesa plots under different values of K are depicted in Figure 6. Compared to Figure 2b, the GLRT-SL experiences a more significant performance loss, with its PD falling below that of G-ABORT-HE. The gap between the detectors is also noticeably smaller. The results in Figure 6 reveal that as K decreases, the selectivity of the proposed GLRT slightly improves.

**Figure 7:**
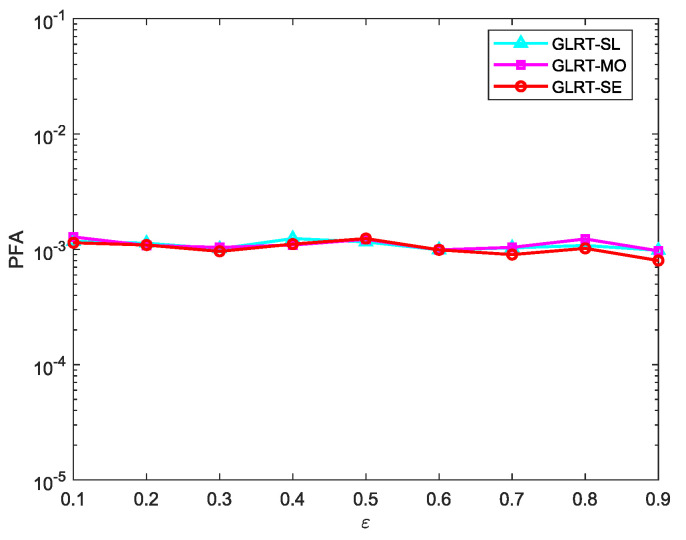
PFA versus ε for N=16, L=2N, and K=4.

Figure 7 shows the CFAR property of the proposed GLRT against the noise covariance matrix. No matter how the correlation coefficient ε changes between 0.1 and 0.9, the PFA hardly changes, which indicates that the proposed GLRT is CFAR.

Table 1 reports the average running time of 20 experiments for both existing and proposed detectors. Specifically, we set $\cos^{2}\theta =1$, SNR=15 dB, and K=2, with the same parameters as above. It is obvious that the GLRT-HE has the shortest running time, while the proposed GLRT has the highest running overhead. This is due to the need to perform a grid search on μ to find the minimum value of (9). Moreover, observing (8), (16), and (17), it is evident that they solely consist of components ${\tilde{X}}^{H}\tilde{X}$ and ${\tilde{X}}^{H}P_{\tilde{h}}^{⊥}\tilde{X}$, and they are all designed based on GLRT. Therefore, the computation complexity among the G-ABORT-HE, GW-ABORT-HE and GLRT-HE is nearly indistinguishable.

## 5. Conclusions

In this paper, we investigate the design of selective detectors for distributed targets embedded in Gaussian noise. The idea is to introduce a fictitious signal under the null hypothesis and model it as a stochastic quantity that follows a complex Gaussian distribution with zero mean and variance of an unknown stochastic factor multiplied by a known rank-one matrix. When signal mismatch occurs, the stochastic fictitious signal captures leaked signals, making the null hypothesis more plausible. Using the GLRT criterion, we derive the corresponding detector and prove its CFAR property. The simulation results reveal that, for example, at SNR=23 dB, the GLRT-SL exhibits the strongest selectivity, even surpassing the GW-ABORT-HE, while at K=2, its detection probability is significantly weaker than G-ABORT-HE in the SNR range of 10 dB to 20 dB. The selectivity of GLRT-SE is weaker than G-ABORT-HE under SNR=23 dB, while it achieves the best performance in matched target detection; in particular, when L=1.5N, the GLRT-SE has the highest detection probability in the range of 13 dB to 20 dB. The behavior of the GLRT-MO lies between these two detectors. Notably, reducing the mismatch between d and the target steering vector enhances selectivity, but at the expense of matched detection performance. Furthermore, the GLRT-SL is superior to the GW-ABORT-HE in both selectivity and matched detection performance, and the GLRT-MO similarly outperforms the G-ABORT-HE. This result demonstrates the benefits of the proposed approach compared to the ABORT-like strategy.

## Figures and Tables

**Table 1:** Computation complexity of the proposed and existing detectors.

Detectors	Running Time(s)
GLRT-HE	23.9290
G-ABORT-HE	24.4529
GW-ABORT-HE	24.1048
Proposed GLRT	32.9585

## Data Availability

The data presented in this study are available upon request from the corresponding author.

## Abbreviations

The following abbreviations are used in this manuscript:

GLRT-HE	the one-step GLRT for Homogeneous Environment
GLRT-SL	the proposed GLRT with slight mismatch d
GLRT-MO	the proposed GLRT with moderate mismatch d
GLRT-SE	the proposed GLRT with severe mismatch d
G-ABORT-HE	Generalized Adaptive Beamformer Orthogonal Rejection Test in Homogeneous Environment
GW-ABORT-HE	Generalized Whitened Adaptive Beamformer Orthogonal Rejection Test in Homogeneous Environment

## Appendix A. Derivation of the GLRT

Let the derivative of (5) with respect to (with respect to.) Σ be zero, and the maximum likelihood estimate (MLE) of Σ under hypothesis $H_{1}$ is

(A1) $${\hat{\Sigma }}_{1}=T^{-1}\left[S+\left(X-ha^{H}\right){\left(X-ha^{H}\right)}^{H}\right]$$

where T=L+K. Plugging (A1) into (5) yields

(A2) $$f_{1}\left(X,Y;{\hat{\Sigma }}_{1}\right)=c{\left|S\right|}^{-T}{\left|I_{K}+{\left(X-ha^{H}\right)}^{H}S^{-1}\left(X-ha^{H}\right)\right|}^{-T}$$

where $c={\left[T/\left(e\pi \right)\right]}^{NT}$. Nulling the derivation of (A2) with respect to. a results in the MLE of a as

(A3) $$\hat{a}=X^{H}S^{-1}h{\left(h^{H}S^{-1}h\right)}^{-1}$$

Plugging (A3) into (A2) leads to

(A4) $$f_{1}\left(X,Y;{\hat{\Sigma }}_{1},\hat{a}\right)=c{\left|S\right|}^{-T}{\left|I_{K}+X^{H}S^{-1}X-X^{H}S^{-1}h{\left(h^{H}S^{-1}h\right)}^{-1}h^{H}S^{-1}X\right|}^{-T}$$

For the convenience of the following deduction, we construct a unitary matrix $D=\left[d,D_{⊥}\right]\in {\mathbb{C}}^{N\times N}$, where $D_{⊥}\in {\mathbb{C}}^{N\times \left(N-1\right)}$ is the orthogonal normalized basis matrix of the orthogonal complementary space ${\langle d\rangle }^{⊥}$ of the vector d. It follows that $d^{H}d=1$, $D_{⊥}^{H}d=0_{\left(N-1\right)\times 1}$ and $D^{H}d=e_{1}={\left[1,0,\cdots ,0\right]}^{T}$ hold true. Further, we define the transformed X and Y as

(A5) $$\tilde{X}=D^{H}X=\left[\begin{array}{c} {\tilde{X}}_{1}\\ {\tilde{X}}_{2} \end{array}\right]$$

and

(A6) $$\tilde{Y}=D^{H}Y=\left[\begin{array}{c} {\tilde{Y}}_{1}\\ {\tilde{Y}}_{2} \end{array}\right]$$

where ${\tilde{X}}_{1}$ and ${\tilde{Y}}_{1}$ are the first rows of $\tilde{X}$ and $\tilde{Y}$, respectively. The transformed Σ is denotes as $\tilde{\Sigma }=D^{H}\Sigma D$. Using $\tilde{X}$, $\tilde{Y}$, and $\tilde{\Sigma }$, (6) can be rewritten as

(A7) $$\begin{array}{cc} f_{0}\left(X,Y\right) & =\pi^{-NT}{\left|D^{H}\Sigma D\right|}^{-L}{\left|D^{H}\left(\Sigma +Pdd^{H}\right)D\right|}^{-K}\mathrm{etr}\left\{-D^{H}\Sigma^{-1}DD^{H}YY^{H}D\right\}\times \mathrm{etr}\left\{-D^{H}{\left(\Sigma +Pdd^{H}\right)}^{-1}DD^{H}XX^{H}D\right\}\\ \; & =\pi^{-NT}{\left|\tilde{\Sigma }\right|}^{-L}{\left|\tilde{\Sigma }+Pe_{1}e_{1}^{H}\right|}^{-K}\mathrm{etr}\left\{-{\tilde{\Sigma }}^{-1}\tilde{Y}{\tilde{Y}}^{H}\right\}\times \mathrm{etr}\left\{-{\left(\tilde{\Sigma }+Pe_{1}e_{1}^{H}\right)}^{-1}\tilde{X}{\tilde{X}}^{H}\right\} \end{array}$$

where the first line of (A7) uses the fact that $\left|D^{H}\Sigma D\right|=\left|D^{H}\right|\left|\Sigma \right|\left|D\right|=\left|\Sigma \right|\left|D^{H}D\right|=\left|\Sigma \right|$, and the second line involves $D^{H}\Sigma^{-1}D=D^{-1}\Sigma^{-1}D={\left(D^{-1}\Sigma D\right)}^{-1}={\left(D^{H}\Sigma D\right)}^{-1}={\tilde{\Sigma }}^{-1}$. Partitioning the matrix $\tilde{\Sigma }$ yields

(A8) $$\tilde{\Sigma }=\left[\begin{array}{cc} {\tilde{\Sigma }}_{11} & {\tilde{\Sigma }}_{12}\\ {\tilde{\Sigma }}_{21} & {\tilde{\Sigma }}_{22} \end{array}\right]=\left[\begin{array}{cc} \underset{1\times 1}{d^{H}\Sigma d} & \underset{1\times \left(N-1\right)}{d^{H}\Sigma D_{⊥}}\\ \underset{\left(N-1\right)\times 1}{D_{⊥}^{H}\Sigma d} & \underset{\left(N-1\right)\times \left(N-1\right)}{D_{⊥}^{H}\Sigma D_{⊥}} \end{array}\right]$$

Since

(A9) $$D_{⊥}^{H}\Sigma D_{⊥}{\left(D_{⊥}^{H}\Sigma D_{⊥}\right)}^{-1}=D_{⊥}^{H}\Sigma D_{⊥}D_{⊥}^{-1}\Sigma^{-1}{\left(D_{⊥}^{H}\right)}^{-1}=D_{⊥}^{H}\Sigma \Sigma^{-1}{\left(D_{⊥}^{H}\right)}^{-1}=D_{⊥}^{H}{\left(D_{⊥}^{H}\right)}^{-1}=D_{⊥}^{H}D_{⊥}=I_{N-1}$$

(A10) $${\left(D_{⊥}^{H}\Sigma D_{⊥}\right)}^{-1}D_{⊥}^{H}\Sigma D_{⊥}=D_{⊥}^{-1}\Sigma^{-1}{\left(D_{⊥}^{H}\right)}^{-1}D_{⊥}^{H}\Sigma D_{⊥}=D_{⊥}^{-1}\Sigma^{-1}\Sigma D_{⊥}=D_{⊥}^{-1}D_{⊥}=I_{N-1}$$

Clearly, the matrix ${\tilde{\Sigma }}_{22}$ is invertible, so the inverse of $\tilde{\Sigma }$ is given by

(A11) $${\tilde{\Sigma }}^{-1}={\left[\begin{array}{cc} {\tilde{\Sigma }}_{11} & {\tilde{\Sigma }}_{12}\\ {\tilde{\Sigma }}_{21} & {\tilde{\Sigma }}_{22} \end{array}\right]}^{-1}={\left[{\tilde{\Sigma }}_{11}-{\tilde{\Sigma }}_{12}{\tilde{\Sigma }}_{22}^{-1}{\tilde{\Sigma }}_{21}\right]}^{-1}\left[\begin{array}{cc} 1 & -{\tilde{\Sigma }}_{12}{\tilde{\Sigma }}_{22}^{-1}\\ -{\tilde{\Sigma }}_{22}^{-1}{\tilde{\Sigma }}_{21} & {\tilde{\Sigma }}_{22}^{-1}{\tilde{\Sigma }}_{21}{\tilde{\Sigma }}_{12}{\tilde{\Sigma }}_{22}^{-1} \end{array}\right]+\left[\begin{array}{cc} 0 & 0\\ 0 & {\tilde{\Sigma }}_{22}^{-1} \end{array}\right]$$

Let ${\tilde{t}}_{21}={\tilde{\Sigma }}_{22}^{-1}{\tilde{\Sigma }}_{21}$, ${\tilde{\Sigma }}_{1\cdot 2}={\tilde{\Sigma }}_{11}-{\tilde{\Sigma }}_{12}{\tilde{\Sigma }}_{22}^{-1}{\tilde{\Sigma }}_{21}$, where ${\tilde{\Sigma }}_{1\cdot 2}$ is the Schur complement of ${\tilde{\Sigma }}_{22}$ in $\tilde{\Sigma }$. Using the fact that ${\tilde{\Sigma }}_{12}={\tilde{\Sigma }}_{21}^{H}$, (A11) can be simplified as

(A12) $${\tilde{\Sigma }}^{-1}={\tilde{\Sigma }}_{1\cdot 2}^{-1}\left[\begin{array}{c} 1\\ -{\tilde{t}}_{21} \end{array}\right]\left[\begin{array}{cc} 1 & -{\tilde{t}}_{21}^{H} \end{array}\right]+\left[\begin{array}{cc} 0 & 0\\ 0 & {\tilde{\Sigma }}_{22}^{-1} \end{array}\right]$$

Similarly, the inverse of $\tilde{\Sigma }+Pe_{1}e_{1}^{H}$ is

(A13) $$\begin{array}{cc} {\left(\tilde{\Sigma }+Pe_{1}e_{1}^{H}\right)}^{-1} & ={\left[\begin{array}{cc} {\tilde{\Sigma }}_{11}+P & {\tilde{\Sigma }}_{12}\\ {\tilde{\Sigma }}_{21} & {\tilde{\Sigma }}_{22} \end{array}\right]}^{-1}\\ \; & ={\left[P+{\tilde{\Sigma }}_{11}-{\tilde{\Sigma }}_{12}{\tilde{\Sigma }}_{22}^{-1}{\tilde{\Sigma }}_{21}\right]}^{-1}\left[\begin{array}{cc} 1 & -{\tilde{\Sigma }}_{12}{\tilde{\Sigma }}_{22}^{-1}\\ -{\tilde{\Sigma }}_{22}^{-1}{\tilde{\Sigma }}_{21} & {\tilde{\Sigma }}_{22}^{-1}{\tilde{\Sigma }}_{21}{\tilde{\Sigma }}_{12}{\tilde{\Sigma }}_{22}^{-1} \end{array}\right]+\left[\begin{array}{cc} 0 & 0\\ 0 & {\tilde{\Sigma }}_{22}^{-1} \end{array}\right]\\ \; & ={\left(P+{\tilde{\Sigma }}_{1.2}\right)}^{-1}\left[\begin{array}{c} 1\\ -{\tilde{t}}_{21} \end{array}\right]\left[\begin{array}{cc} 1 & -{\tilde{t}}_{21}^{H} \end{array}\right]+\left[\begin{array}{cc} 0 & 0\\ 0 & {\tilde{\Sigma }}_{22}^{-1} \end{array}\right] \end{array}$$

Observing (A11)–(A13), the unknown parameter set under the hypothesis $H_{0}$ can be equivalently represented as $\Theta_{0}=\left(P,{\tilde{\Sigma }}_{22},{\tilde{t}}_{21},{\tilde{\Sigma }}_{1\cdot 2}\right)$. Furthermore, using the partitioned matrix determinant lemma, we obtain

(A14) $$\left|\tilde{\Sigma }\right|=\left|\begin{array}{cc} {\tilde{\Sigma }}_{11} & {\tilde{\Sigma }}_{12}\\ {\tilde{\Sigma }}_{21} & {\tilde{\Sigma }}_{22} \end{array}\right|=\left|{\tilde{\Sigma }}_{22}\right|\left({\tilde{\Sigma }}_{11}-{\tilde{\Sigma }}_{12}{\tilde{\Sigma }}_{22}^{-1}{\tilde{\Sigma }}_{21}\right)={\tilde{\Sigma }}_{1\cdot 2}\left|{\tilde{\Sigma }}_{22}\right|$$

According to (A7), (A12)–(A14) can be recast as

(A15) $$\begin{array}{cc} f_{0}\left(X,Y\right) & =\pi^{-NT}{\left|\tilde{\Sigma }\right|}^{-L}{\left|\tilde{\Sigma }\right|}^{-K}{\left(1+Pe_{1}^{H}{\tilde{\Sigma }}^{-1}e_{1}\right)}^{-K}\times \mathrm{etr}\left\{-{\tilde{Y}}^{H}{\tilde{\Sigma }}^{-1}\tilde{Y}\right\}\times \mathrm{etr}\left\{-{\tilde{X}}^{H}{\left(\tilde{\Sigma }+Pe_{1}e_{1}^{H}\right)}^{-1}\tilde{X}\right\}\\ \; & =\pi^{-NT}{\tilde{\Sigma }}_{1\cdot 2}^{-T}{\left|{\tilde{\Sigma }}_{22}\right|}^{-T}{\left[{\tilde{\Sigma }}_{1\cdot 2}^{-1}\left(P+{\tilde{\Sigma }}_{1\cdot 2}\right)\right]}^{-K}\times \mathrm{etr}\left\{-\left[\begin{array}{cc} {\tilde{Y}}_{1}^{H} & {\tilde{Y}}_{2}^{H} \end{array}\right]\left[\begin{array}{cc} 0 & 0\\ 0 & {\tilde{\Sigma }}_{22}^{-1} \end{array}\right]\left[\begin{array}{c} {\tilde{Y}}_{1}\\ {\tilde{Y}}_{2} \end{array}\right]-{\tilde{Y}}^{H}\left\{{\tilde{\Sigma }}_{1\cdot 2}^{-1}\left[\begin{array}{c} 1\\ -{\tilde{t}}_{21} \end{array}\right]\left[\begin{array}{cc} 1 & -{\tilde{t}}_{21}^{H} \end{array}\right]\right\}\tilde{Y}\right\}\\ \; & =\pi^{-NT}{\left|\tilde{\Sigma }\right|}^{-T}{\left(1+P{\tilde{\Sigma }}_{1\cdot 2}^{-1}\right)}^{-K}\mathrm{etr}\left\{-\left[\begin{array}{cc} {\tilde{X}}_{1}^{H} & {\tilde{X}}_{2}^{H} \end{array}\right]\left[\begin{array}{cc} 0 & 0\\ 0 & {\tilde{\Sigma }}_{22}^{-1} \end{array}\right]\left[\begin{array}{c} {\tilde{X}}_{1}\\ {\tilde{X}}_{2} \end{array}\right]-{\tilde{X}}^{H}\left\{{\left(P+{\tilde{\Sigma }}_{1\cdot 2}\right)}^{-1}\left[\begin{array}{c} 1\\ -{\tilde{t}}_{21} \end{array}\right]\left[\begin{array}{cc} 1 & -{\tilde{t}}_{21}^{H} \end{array}\right]\right\}\tilde{X}\right\}\\ \; & \times \mathrm{etr}\left\{-{\tilde{X}}^{H}\left\{{\left(P+{\tilde{\Sigma }}_{1\cdot 2}\right)}^{-1}\left[\begin{array}{c} 1\\ -{\tilde{t}}_{21} \end{array}\right]\left[\begin{array}{cc} 1 & -{\tilde{t}}_{21}^{H} \end{array}\right]+\left[\begin{array}{cc} 0 & 0\\ 0 & {\tilde{\Sigma }}_{22}^{-1} \end{array}\right]\right\}\tilde{X}\right\}\mathrm{etr}\left\{-{\tilde{Y}}^{H}\left\{{\tilde{\Sigma }}_{1\cdot 2}^{-1}\left[\begin{array}{c} 1\\ -{\tilde{t}}_{21} \end{array}\right]\left[\begin{array}{cc} 1 & -{\tilde{t}}_{21}^{H} \end{array}\right]+\left[\begin{array}{cc} 0 & 0\\ 0 & {\tilde{\Sigma }}_{22}^{-1} \end{array}\right]\right\}\tilde{Y}\right\}\\ \; & =\pi^{-NT}{\tilde{\Sigma }}_{1\cdot 2}^{-L}{\left|{\tilde{\Sigma }}_{22}\right|}^{-T}{\left(P+{\tilde{\Sigma }}_{1\cdot 2}\right)}^{-K}\times \mathrm{etr}\left\{-{\tilde{Y}}_{2}^{H}{\tilde{\Sigma }}_{22}^{-1}{\tilde{Y}}_{2}-{\tilde{Y}}^{H}\left\{{\tilde{\Sigma }}_{1\cdot 2}^{-1}\left[\begin{array}{c} 1\\ -{\tilde{t}}_{21} \end{array}\right]\left[\begin{array}{cc} 1 & -{\tilde{t}}_{21}^{H} \end{array}\right]\right\}\tilde{Y}\right\}\\ \; & \times \mathrm{etr}\left\{-{\tilde{X}}_{2}^{H}{\tilde{\Sigma }}_{22}^{-1}{\tilde{X}}_{2}-{\tilde{X}}^{H}\left\{{\left(P+{\tilde{\Sigma }}_{1\cdot 2}\right)}^{-1}\left[\begin{array}{c} 1\\ -{\tilde{t}}_{21} \end{array}\right]\left[\begin{array}{cc} 1 & -{\tilde{t}}_{21}^{H} \end{array}\right]\right\}\tilde{X}\right\}\\ \; & =\pi^{-NT}{\tilde{\Sigma }}_{1\cdot 2}^{-L}{\left(P+{\tilde{\Sigma }}_{1\cdot 2}\right)}^{-K}{\left|{\tilde{\Sigma }}_{22}\right|}^{-T}\mathrm{etr}\left\{-{\tilde{\Sigma }}_{22}^{-1}\left({\tilde{X}}_{2}{\tilde{X}}_{2}^{H}+{\tilde{Y}}_{2}{\tilde{Y}}_{2}^{H}\right)\right\}\times \exp\left\{-\left[\begin{array}{cc} 1 & -{\tilde{t}}_{21}^{H} \end{array}\right]\left\{{\left(P+{\tilde{\Sigma }}_{1\cdot 2}\right)}^{-1}\tilde{X}{\tilde{X}}^{H}+{\tilde{\Sigma }}_{1\cdot 2}^{-1}\tilde{Y}{\tilde{Y}}^{H}\right\}\left[\begin{array}{c} 1\\ -{\tilde{t}}_{21} \end{array}\right]\right\} \end{array}$$

For convenience, define a temporary term $\tilde{A}$ as

(A16) $$\tilde{A}={\left(P+{\tilde{\Sigma }}_{1\cdot 2}\right)}^{-1}\tilde{X}{\tilde{X}}^{H}+{\tilde{\Sigma }}_{1\cdot 2}^{-1}\tilde{S}$$

where $\tilde{S}=\tilde{Y}{\tilde{Y}}^{H}$. Plugging (A16) into (A15) results in

(A17) $$f_{0}\left(X,Y\right)=\pi^{-NT}{\tilde{\Sigma }}_{1\cdot 2}^{-L}{\left(P+{\tilde{\Sigma }}_{1\cdot 2}\right)}^{-K}{\left|{\tilde{\Sigma }}_{22}\right|}^{-T}\mathrm{etr}\left\{-{\tilde{\Sigma }}_{22}^{-1}\left({\tilde{X}}_{2}{\tilde{X}}_{2}^{H}+{\tilde{Y}}_{2}{\tilde{Y}}_{2}^{H}\right)\right\}\exp\left\{-\left[\begin{array}{cc} 1 & -{\tilde{t}}_{21}^{H} \end{array}\right]\tilde{A}\left[\begin{array}{c} 1\\ -{\tilde{t}}_{21} \end{array}\right]\right\}$$

Partitioning $\tilde{A}$ yields

(A18) $$\tilde{A}=\left[\begin{array}{cc} \underset{1\times 1}{{\tilde{A}}_{11}} & \underset{1\times \left(N-1\right)}{{\tilde{A}}_{12}}\\ \underset{\left(N-1\right)\times 1}{{\tilde{A}}_{21}} & \underset{\left(N-1\right)\times \left(N-1\right)}{{\tilde{A}}_{22}} \end{array}\right]$$

According to (A18), the following equation holds true

(A19) $$\begin{array}{cc} \left[\begin{array}{cc} 1 & -{\tilde{t}}_{21}^{H} \end{array}\right]\tilde{A}\left[\begin{array}{c} 1\\ -{\tilde{t}}_{21} \end{array}\right] & ={\tilde{A}}_{11}-{\tilde{A}}_{12}{\tilde{A}}_{22}{\tilde{A}}_{22}^{-1}{\tilde{t}}_{21}-{\tilde{t}}_{21}^{H}{\tilde{A}}_{22}{\tilde{A}}_{22}^{-1}{\tilde{A}}_{21}+{\tilde{t}}_{21}^{H}{\tilde{A}}_{22}{\tilde{t}}_{21}+{\tilde{A}}_{12}{\tilde{A}}_{22}^{-1}{\tilde{A}}_{21}-{\tilde{A}}_{12}{\tilde{A}}_{22}^{-1}{\tilde{A}}_{21}\\ \; & ={\tilde{A}}_{1\cdot 2}+{\left({\tilde{t}}_{21}-{\tilde{A}}_{22}^{-1}{\tilde{A}}_{21}\right)}^{H}{\tilde{A}}_{22}\left({\tilde{t}}_{21}-{\tilde{A}}_{22}^{-1}{\tilde{A}}_{21}\right) \end{array}$$

where ${\tilde{A}}_{1\cdot 2}$ is the Schur complement of ${\tilde{A}}_{22}$ in $\tilde{A}$. Plugging (A19) into (A17) leads to

(A20) $$f_{0}\left(X,Y\right)=\pi^{-NT}{\tilde{\Sigma }}_{1\cdot 2}^{-L}{\left(P+{\tilde{\Sigma }}_{1\cdot 2}\right)}^{-K}{\left|{\tilde{\Sigma }}_{22}\right|}^{-T}\mathrm{etr}\left\{-{\tilde{\Sigma }}_{22}^{-1}\left({\tilde{X}}_{2}{\tilde{X}}_{2}^{H}+{\tilde{Y}}_{2}{\tilde{Y}}_{2}^{H}\right)\right\}\exp\left\{-{\tilde{A}}_{1\cdot 2}\right\}\exp\left\{-{\left({\tilde{t}}_{21}-{\tilde{A}}_{22}^{-1}{\tilde{A}}_{21}\right)}^{H}{\tilde{A}}_{22}\left({\tilde{t}}_{21}-{\tilde{A}}_{22}^{-1}{\tilde{A}}_{21}\right)\right\}$$

Nulling the derivation of (A20) with respect to ${\tilde{\Sigma }}_{22}$ yields

(A21) $${\hat{\tilde{\Sigma }}}_{22}=T^{-1}\left({\tilde{X}}_{2}{\tilde{X}}_{2}^{H}+{\tilde{Y}}_{2}{\tilde{Y}}_{2}^{H}\right)$$

Plugging (A21) into (A20), after some algebra, results in

(A22) $$f_{0}\left(X,Y;{\hat{\tilde{\Sigma }}}_{22}\right)={\left(T/e\pi \right)}^{NT}{\tilde{\Sigma }}_{1\cdot 2}^{-L}{\left(P+{\tilde{\Sigma }}_{1\cdot 2}\right)}^{-K}{\left|{\tilde{X}}_{2}{\tilde{X}}_{2}^{H}+{\tilde{Y}}_{2}{\tilde{Y}}_{2}^{H}\right|}^{-T}\times \exp\left\{-{\tilde{A}}_{1\cdot 2}\right\}\times \exp\left\{-{\left({\tilde{t}}_{21}-{\tilde{A}}_{22}^{-1}{\tilde{A}}_{21}\right)}^{H}{\tilde{A}}_{22}\left({\tilde{t}}_{21}-{\tilde{A}}_{22}^{-1}{\tilde{A}}_{21}\right)\right\}$$

Then, nulling the derivation of (A22) with respect to ${\tilde{t}}_{21}$ leads to

(A23) $${\hat{\tilde{t}}}_{21}={\tilde{A}}_{22}^{-1}{\tilde{A}}_{21}$$

Plugging (A23) into (A22), after some algebra, yields

(A24) $$f_{0}\left(X,Y;{\hat{\tilde{\Sigma }}}_{22},{\hat{\tilde{t}}}_{21}\right)={\left(T/e\pi \right)}^{NT}{\tilde{\Sigma }}_{1\cdot 2}^{-L}{\left(P+{\tilde{\Sigma }}_{1\cdot 2}\right)}^{-K}{\left|{\tilde{X}}_{2}{\tilde{X}}_{2}^{H}+{\tilde{Y}}_{2}{\tilde{Y}}_{2}^{H}\right|}^{-T}\times \exp\left\{-{\;\tilde{A}}_{1\cdot 2}\right\}$$

Using the fact that

(A25) $${\tilde{A}}^{-1}={\left\{{\tilde{\Sigma }}_{1\cdot 2}^{-1}\left[{\left(P+{\tilde{\Sigma }}_{1\cdot 2}\right)}^{-1}{\tilde{\Sigma }}_{1\cdot 2}\tilde{X}{\tilde{X}}^{H}+\tilde{S}\right]\right\}}^{-1}={\tilde{\Sigma }}_{1\cdot 2}D^{H}{\left[S+{\left(1+P{\tilde{\Sigma }}_{1\cdot 2}^{-1}\right)}^{-1}XX^{H}\right]}^{-1}D$$

We can easily obtain that the first block of ${\tilde{A}}^{-1}$ is

(A26) $${\tilde{A}}_{1\cdot 2}^{-1}={\tilde{\Sigma }}_{1\cdot 2}d^{H}{\left[S+{\left(1+P{\tilde{\Sigma }}_{1\cdot 2}^{-1}\right)}^{-1}XX^{H}\right]}^{-1}d$$

Similarly, the first block of ${\tilde{\Sigma }}^{-1}$ can be obtained by

(A27) $${\tilde{\Sigma }}^{-1}={\left(D^{H}\Sigma D\right)}^{-1}=D^{H}\Sigma^{-1}D=\left[\begin{array}{cc} d^{H}\Sigma^{-1}d & d^{H}\Sigma^{-1}D_{⊥}\\ D_{⊥}^{H}\Sigma^{-1}d & D_{⊥}^{H}\Sigma^{-1}D_{⊥} \end{array}\right]=\left[\begin{array}{cc} {\tilde{\Sigma }}_{1\cdot 2}^{-1} & \times \\ \times & \times \end{array}\right]$$

Let $a={\tilde{\Sigma }}_{1\cdot 2}={\left(d^{H}\Sigma^{-1}d\right)}^{-1}>0$, $\mu ={\left(1+P{\tilde{\Sigma }}_{1\cdot 2}^{-1}\right)}^{-1}={\left(1+Pd^{H}\Sigma^{-1}d\right)}^{-1}$, (A24) can be recast as

(A28) $$\begin{array}{cc} f_{0}\left(X,Y;{\hat{\tilde{\Sigma }}}_{22},{\hat{\tilde{t}}}_{21}\right) & ={\left(T/e\pi \right)}^{NT}{\left(1+P{\tilde{\Sigma }}_{1\cdot 2}^{-1}\right)}^{-K}{\left({\tilde{\Sigma }}_{1\cdot 2}\right)}^{-K}{\left|{\tilde{\Sigma }}_{1\cdot 2}\right|}^{-L}{\left|{\tilde{X}}_{2}{\tilde{X}}_{2}^{H}+{\tilde{Y}}_{2}{\tilde{Y}}_{2}^{H}\right|}^{-T}\times \exp\left\{-a^{-1}{\left[d^{H}{\left(S+{\left(1+b\right)}^{-1}XX^{H}\right)}^{-1}d\right]}^{-1}\right\}\\ \; & ={\left(T/e\pi \right)}^{NT}a^{-T}\mu^{-K}{\left|D_{⊥}^{H}\left(S+XX^{H}\right)D_{⊥}\right|}^{-T}\exp\left\{-a^{-1}{\left[d^{H}{\left(S+\mu XX^{H}\right)}^{-1}d\right]}^{-1}\right\} \end{array}$$

Then, let function $h\left(a\right)=a^{-T}\exp\left(-l^{-1}a^{-1}\right)$, where $l=d^{H}{\left[S+\mu XX^{H}\right]}^{-1}d>0$. The derivation of the logarithm of $h\left(a\right)$ is

(A29) $$\frac{\partial \ln h\left(a\right)}{\partial a}=\frac{-aT+l^{-1}}{a^{2}}$$

It is readily apparent that $h\left(a\right)$ attains its maximum value at $a={\left(Tl\right)}^{-1}$, and we have

(A30) $$h{\left(a\right)}_{\max}={\left(T/e\right)}^{T}{\left[d^{H}{\left(S+\mu XX^{H}\right)}^{-1}d\right]}^{T}$$

Plugging (A30) into (A28) leads to

(A31) $$f_{0}\left(X,Y;{\hat{\tilde{\Sigma }}}_{22},{\hat{\tilde{t}}}_{21},\hat{a}\right)={\left(T/e\right)}^{\left(N+1\right)T}\pi^{-NT}\mu^{-K}{\left|D_{⊥}^{H}\left(S+XX^{H}\right)D_{⊥}\right|}^{-T}{\left[d^{H}{\left(S+\mu XX^{H}\right)}^{-1}d\right]}^{T}$$

Utilizing the matrix determinant lemma, the subsequent equations hold true

(A32) $$\begin{array}{cc} \left|D_{⊥}^{H}\left(S+\mu XX^{H}\right)D_{⊥}\right| & =\left|D_{⊥}^{H}SD_{⊥}\right|\left|I_{K}+\mu X^{H}D_{⊥}{\left(D_{⊥}^{H}SD_{⊥}\right)}^{-1}D_{⊥}^{H}X\right|\\ \; & =\left|D_{⊥}^{H}SD_{⊥}\right|\left|I_{K}+\mu X^{H}S^{-1/2}S^{1/2}D_{⊥}{\left(D_{⊥}^{H}S^{1/2}S^{1/2}D_{⊥}\right)}^{-1}D_{⊥}^{H}X\right|\\ \; & =\left|D_{⊥}^{H}SD_{⊥}\right|\left|I_{K}+\mu X^{H}S^{-1/2}P_{S^{1/2}D_{⊥}}S^{-1/2}X\right|\\ \; & =\left|D_{⊥}^{H}SD_{⊥}\right|\left|I_{K}+\mu X^{H}S^{-1/2}P_{S^{-1/2}d}^{⊥}S^{-1/2}X\right| \end{array}$$

(A33) $$\begin{array}{cc} \left|I_{K}+\mu X^{H}S^{-1/2}P_{S^{-1/2}d}^{⊥}S^{-1/2}X\right| & =\left|I_{K}+\mu X^{H}S^{-1/2}\left(I_{N}-P_{S^{-1/2}d}\right)S^{-1/2}X\right|\\ \; & =\left|I_{K}+\mu X^{H}S^{-1}X-\mu X^{H}S^{-1/2}P_{S^{-1/2}d}S^{-1/2}X\right|\\ \; & =\left|I_{K}+\mu X^{H}S^{-1}X-\mu \frac{X^{H}S^{-1}dd^{H}S^{-1}X}{d^{H}S^{-1}d}\right|\\ \; & =\left|I_{K}+\mu X^{H}S^{-1}X\right|\left[1-\mu \frac{d^{H}S^{-1}X{\left(I_{K}+\delta X^{H}S^{-1}X\right)}^{-1}X^{H}S^{-1}d}{d^{H}S^{-1}d}\right]\\ \; & =\left|I_{K}+\mu X^{H}S^{-1}X\right|\times \frac{d^{H}\left[S^{-1}-\mu S^{-1}X{\left(I_{K}+\mu X^{H}S^{-1}X\right)}^{-1}X^{H}S^{-1}\right]d}{d^{H}S^{-1}d}\\ \; & =\left|I_{K}+\mu X^{H}S^{-1}X\right|\times \frac{d^{H}{\left(S+\mu XX^{H}\right)}^{-1}d}{d^{H}S^{-1}d} \end{array}$$

(A34) $$\begin{array}{cc} \left|S\right| & =\left|D^{H}SD\right|\\ \; & =\left|D_{⊥}^{H}SD_{⊥}\right|\left[d^{H}Sd-d^{H}SD_{⊥}{\left(D_{⊥}^{H}SD_{⊥}\right)}^{-1}D_{⊥}^{H}Sd\right]\\ \; & =\left|D_{⊥}^{H}SD_{⊥}\right|\left[d^{H}S^{1/2}I_{N}S^{1/2}d-d^{H}S^{1/2}S^{1/2}D_{⊥}{\left(D_{⊥}^{H}S^{1/2}S^{1/2}D_{⊥}\right)}^{-1}D_{⊥}^{H}S^{1/2}S^{1/2}d\right]\\ \; & =\left|D_{⊥}^{H}SD_{⊥}\right|\left[d^{H}S^{1/2}\left(I_{N}-P_{S^{1/2}D_{⊥}}\right)S^{1/2}d\right]\\ \; & =\left|D_{⊥}^{H}SD_{⊥}\right|\left[d^{H}S^{1/2}P_{S^{-1/2}d}S^{1/2}d\right]\\ \; & =\left|D_{⊥}^{H}SD_{⊥}\right|{\left(d^{H}S^{-1}d\right)}^{-1} \end{array}$$

Let μ=1 and plugging (A33) and (A34) into (A32) yields

(A35) $$\left|D_{⊥}^{H}\left(S+XX^{H}\right)D_{⊥}\right|=\left|S\right|\left|I_{K}+X^{H}S^{-1}X\right|d^{H}{\left(S+XX^{H}\right)}^{-1}d$$

Plugging (A35) into (A31) results in

(A36) $$f_{0}\left(X,Y;{\hat{\tilde{\Sigma }}}_{22},{\hat{\tilde{t}}}_{21},\hat{a}\right)={\left(T/e\right)}^{\left(N+1\right)T}\pi^{-NT}\mu^{-K}{\left|S\right|}^{-T}{\left|I_{K}+X^{H}S^{-1}X\right|}^{-T}\times {\left[\frac{d^{H}{\left(S+\mu XX^{H}\right)}^{-1}d}{d^{H}{\left(S+XX^{H}\right)}^{-1}d}\right]}^{T}$$

Taking the Tth root of the ratio of (A4) and (A36), and omit the constant term to obtain (7). According to (A35), we have

(A37) $$d^{H}{\left(S+\mu XX^{H}\right)}^{-1}d=\frac{\left|I_{K}+\mu X^{H}S^{-1/2}P_{S^{-1/2}d}^{⊥}S^{-1/2}X\right|}{\left|I_{K}+\mu X^{H}S^{-1}X\right|}\times d^{H}S^{-1}d$$

Plugging (A34) and (A37) into (A31) leads to

(A38) $$f_{0}\left(X,Y;{\hat{\tilde{\Sigma }}}_{22},{\hat{\tilde{t}}}_{21},\hat{a}\right)={\left(T/e\right)}^{\left(N+1\right)T}\pi^{-NT}\mu^{-K}{\left|S\right|}^{-T}{\left|I_{K}+X^{H}S^{-1/2}P_{S^{-1/2}d}^{⊥}S^{-1/2}X\right|}^{-T}\times \frac{{\left|I_{K}+\mu X^{H}S^{-1/2}P_{S^{-1/2}d}^{⊥}S^{-1/2}X\right|}^{T}}{{\left|I_{K}+\mu X^{H}S^{-1}X\right|}^{T}}$$

Taking the Tth root of the ratio of (A4) and (A38), and omit the constant term to obtain (10).

## Appendix B. Proof of the CFAR Property of the GLRT

As the GLRT-HE has been shown to possess the CFAR property [11], it is only necessary to prove that the statistical distribution of the correction term of the proposed GLRT under the null hypothesis is independent of the noise covariance matrix Σ. For convenience, let

(A39) $$\Lambda =\frac{d^{H}{\left(S_{x}+S_{y}\right)}^{-1}d}{d^{H}{\left(\mu S_{x}+S_{y}\right)}^{-1}d}$$

where $S_{x}=XX^{H}$, $S_{y}=S$. The quantity Λ can be further converted to

(A40) $$\Lambda =\frac{d^{H}\Sigma^{-1/2}{\left(\Sigma^{-1/2}S_{x}\Sigma^{-1/2}+\Sigma^{-1/2}S_{y}\Sigma^{-1/2}\right)}^{-1}\Sigma^{-1/2}d}{d^{H}\Sigma^{-1/2}{\left(\mu \Sigma^{-1/2}S_{x}\Sigma^{-1/2}+\Sigma^{-1/2}S_{y}\Sigma^{-1/2}\right)}^{-1}\Sigma^{-1/2}d}=\frac{{\bar{d}}^{H}{\left({\bar{S}}_{x}+{\bar{S}}_{y}\right)}^{-1}\bar{d}}{{\bar{d}}^{H}{\left(\mu {\bar{S}}_{x}+{\bar{S}}_{y}\right)}^{-1}\bar{d}}$$

where $\bar{d}=\Sigma^{-1/2}d$, ${\bar{S}}_{x}=\Sigma^{-1/2}S_{x}\Sigma^{-1/2}$, and ${\bar{S}}_{y}=\Sigma^{-1/2}S_{y}\Sigma^{-1/2}$. According to the statistical distribution of X under $H_{0}$ in (3), the following distribution holds true

(A41) $$\Sigma^{-1/2}X\sim CN_{N,K}\left(0,I_{N}+P\Sigma^{-1/2}dd^{H}\Sigma^{-1/2},I_{K}\right)$$

It is straightforward to demonstrate that the distribution of ${\bar{S}}_{x}$ under hypothesis $H_{0}$ is

(A42) $${\bar{S}}_{x}=\Sigma^{-1/2}XX^{H}\Sigma^{-1/2}\sim CW_{N}\left(K,I_{N}+P\Sigma^{-1/2}dd^{H}\Sigma^{-1/2}\right)$$

Similarly, the distribution of ${\bar{S}}_{y}$ under hypothesis $H_{0}$ is

(A43) $${\bar{S}}_{y}=\Sigma^{-1/2}S\Sigma^{-1/2}\sim CW_{N}\left(L,I_{N}\right)$$

Without loss of generality, we define a unitary matrix $U=\left[\bar{d}/\left\|\bar{d}\right\|,U_{1}\right]\in {\mathbb{C}}^{N\times N}$, where $U_{1}\in {\mathbb{C}}^{N\times \left(N-1\right)}$ denotes a semi-unitary matrix, ${\left(\bar{d}/\left\|\bar{d}\right\|\right)}^{H}\bar{d}/\left\|\bar{d}\right\|=e_{1}$ and $U_{1}^{H}\bar{d}/\left\|\bar{d}\right\|=0_{\left(N-1\right)\times 1}$ hold true. It follows that the fact that $U^{H}\bar{d}={\left(d^{H}\Sigma^{-1}d\right)}^{1/2}e_{1}$ also holds true. Then, the unitary matrix transformation of the quantities in Λ using U yields

(A44) $$\Lambda =\frac{{\bar{d}}^{H}U{\left(U^{H}{\bar{S}}_{x}U+U^{H}{\bar{S}}_{y}U\right)}^{-1}U^{H}\bar{d}}{{\bar{d}}^{H}U{\left(\mu U^{H}{\bar{S}}_{x}U+U^{H}{\bar{S}}_{y}U\right)}^{-1}U^{H}\bar{d}}=\frac{e_{1}^{H}{\left({\bar{S}}_{Ux}+{\bar{S}}_{Uy}\right)}^{-1}e_{1}}{e_{1}^{H}{\left(\mu {\bar{S}}_{Ux}+{\bar{S}}_{Uy}\right)}^{-1}e_{1}}$$

where ${\bar{S}}_{Ux}=U^{H}{\bar{S}}_{x}U$, and ${\bar{S}}_{Uy}=U^{H}{\bar{S}}_{y}U$. It is readily verifiable that

(A45) $$\begin{array}{cc} {\bar{S}}_{Ux} & =U^{H}{\bar{S}}_{x}U\sim CW_{N}\left(K,U^{H}I_{N}U+PU^{H}\Sigma^{-1/2}dd^{H}\Sigma^{-1/2}U\right)\\ \; & =CW_{N}\left(K,I_{N}+PU^{H}\bar{d}{\bar{d}}^{H}U\right)\\ \; & =CW_{N}\left(K,I_{N}+P{\left(d^{H}\Sigma^{-1}d\right)}^{1/2}e_{1}e_{1}^{H}{\left(d^{H}\Sigma^{-1}d\right)}^{1/2}\right)\\ \; & =CW_{N}\left(K,I_{N}+Pd^{H}\Sigma^{-1}de_{1}e_{1}^{H}\right)\\ \; & =CW_{N}\left(K,I_{N}+\left(\mu^{-1}-1\right)e_{1}e_{1}^{H}\right) \end{array}$$

and

(A46) $${\bar{S}}_{Uy}=U^{H}{\bar{S}}_{y}U\sim CW_{N}\left(L,I_{N}\right)$$

Obviously, the distribution of Λ under hypothesis $H_{0}$ is independent of Σ. Hence, the correction term of the proposed GLRT is a stochastic variable whose statistical distribution does not depend on Σ, i.e., the proposed GLRT possesses the CFAR property.
